# SperMD: the expression atlas of sperm maturation

**DOI:** 10.1186/s12859-024-05631-x

**Published:** 2024-01-17

**Authors:** Yifan Li, Qianying Li, Lvying Wu, Haiyan Wang, Hui Shi, Chenhui Yang, Yiqun Gu, Jianyuan Li, Zhiliang Ji

**Affiliations:** 1 School of Informatics, National Institute for Data Science in Health and Medicine, Xiamen University, Xiamen, China; 2 State Key Laboratory of Cellular Stress Biology, School of Life Sciences, Faculty of Medicine and Life Sciences, Xiamen University, Xiamen, China; 3 Shandong Epihealth Biotech Ltd., Yantai, China; 4 College of Life Science, Yantai University, Yantai, China; 5 Institute of science and technology, National Health Commission, Beijing, China

**Keywords:** Database, Multi-omics, Data integration, Sperm maturation, Male subfertility

## Abstract

The impairment of sperm maturation is one of the major pathogenic factors in male subfertility, a serious medical and social problem affecting millions of global couples. Regrettably, the existing research on sperm maturation is slow, limited, and fragmented, largely attributable to the lack of a global molecular view. To fill the data gap, we newly established a database, namely the Sperm Maturation Database (SperMD, http://bio-add.org/SperMD). SperMD integrates heterogeneous multi-omics data (170 transcriptomes, 91 proteomes, and five human metabolomes) to illustrate the transcriptional, translational, and metabolic manifestations during the entire lifespan of sperm maturation. These data involve almost all crucial scenarios related to sperm maturation, including the tissue components of the epididymal microenvironment, cell constituents of tissues, different pathological states, and so on. To the best of our knowledge, SperMD could be one of the limited repositories that provide focused and comprehensive information on sperm maturation. Easy-to-use web services are also implemented to enhance the experience of data retrieval and molecular comparison between humans and mice. Furthermore, the manuscript illustrates an example application demonstrated to systematically characterize novel gene functions in sperm maturation. Nevertheless, SperMD undertakes the endeavor to integrate the islanding omics data, offering a panoramic molecular view of how the spermatozoa gain full reproductive abilities. It will serve as a valuable resource for the systematic exploration of sperm maturation and for prioritizing the biomarkers and targets for precise diagnosis and therapy of male subfertility.

**Supplementary Information:**

The online version contains supplementary material available at 10.1186/s12859-024-05631-x.

## Background

Approximately 12–15% of global couples are suffering from infertility problems, affecting more than 186 million individuals [1], therein up to 50% of the problems can be attributable to men [2]. More disquieting, the human sperm quality has manifested a downward trend in recent decades [3, 4], indicating that subfertility has become a risk factor for the whole human beings. Regretfully, current clinical practices like semen analysis often fail to precisely diagnose the cause of male subfertility and consequently, the husbands have to bear the treatment with uncertainty [2, 5, 6]. Male infertility and subfertility are pathologically diverse, about 60–70% of pathogenesis still remains unknown even though the semen analysis has been carefully demonstrated [7]. It was estimated that up to 40% of infertile males could have idiopathic infertility related to sperm maturational disorders [8]. Therefore, there is an urgent need for a deeper and more thorough investigation into the research on how sperm can be fertile.

Sperm maturation is the critical process for the spermatozoa to acquire natural fertilizing capacities such as motility, capacitation, acrosome reaction, egg penetration, and so on through the epididymis. For years, extensive investigations were conducted to link sperm functions with male infertility [9]. These efforts achieved significant advances in unveiling various active roles of the epididymis in post-testicular modifications of spermatozoa. In recent years, wide applications of omics technologies, particularly the sequencing and mass spectrum technologies, further broaden the scope of the epididymis and sperm research to a vantage that the gene/protein behaviors can be monitored on a large scale. These attempts provide new insights into sperm maturation from multiple views, for instance, the Mammalian Reproductive Genetics Database V2 (MRGDv2) amassed 988 published RNA-seq datasets, including the reproductive and non-reproductive tissues of both males and females in human and mice [10]. the SpermBase deposited transcriptome data of both mRNAs and small RNAs determined in the human, mouse, rat, and rabbit spermatozoa [11]. The REPRODUCTION-2DPAGE (http://reprod.njmu.edu.cn/2d) collected 2D-gel maps and LC-MS/MS-based proteome data of both male and female reproductive tissues for humans and mice [12]. Notably, all these databases are not specially developed for sperm maturation research. The MRGDv2 database concerns a broad range of reproductive biology, and the other two databases mainly focus on spermatogenesis. Still, many multi-omics data just scattered in several public repositories such as the Human Protein Atlas [13], the Expression Atlas [14], and the Human Metabolome Database (HMDB) [15]. These data were determined for different biological purposes rather than sperm maturation; however, they contain valuable information on epididymis and spermatozoa which should be but hasn’t been properly used.

**Fig. 1:**
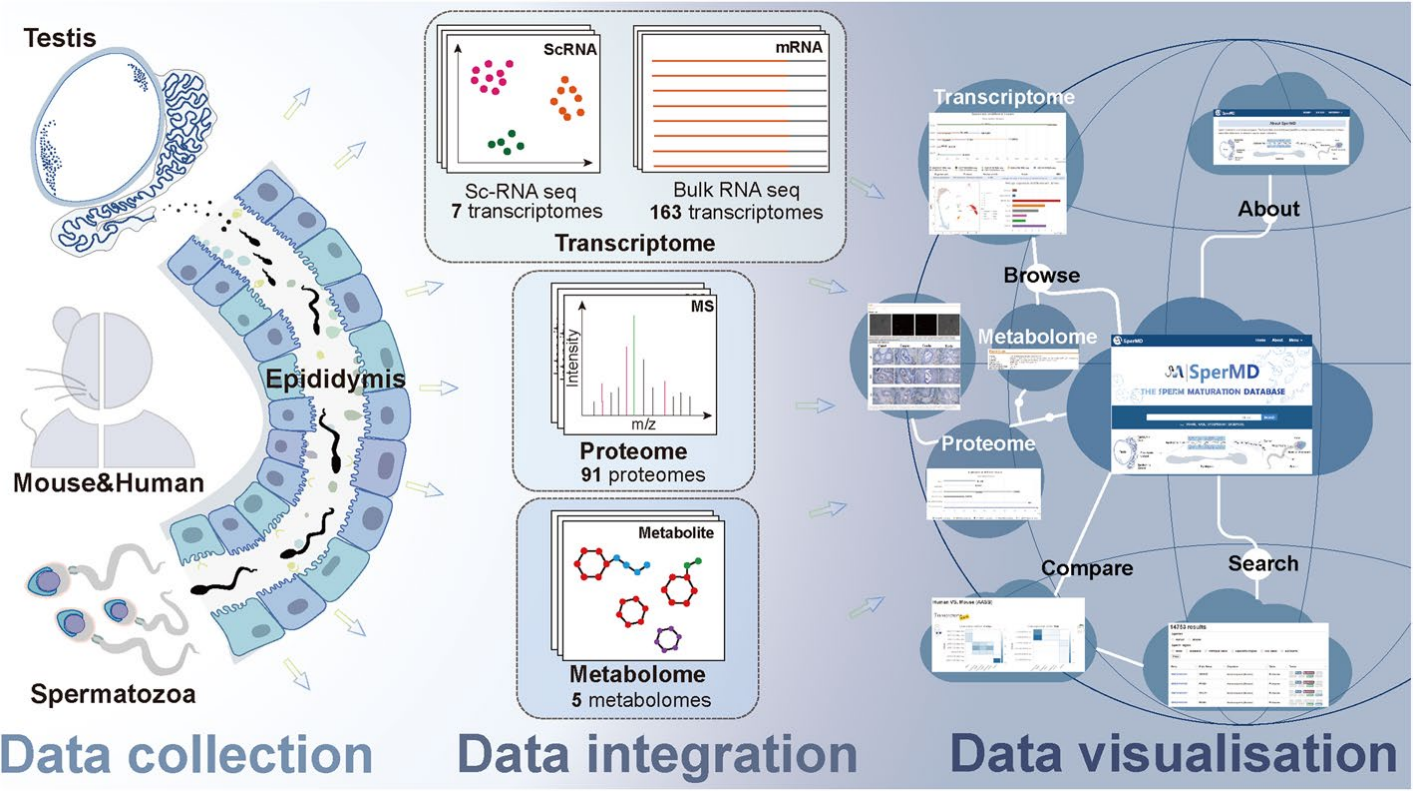
The schematic illustration of constructing the SperMD database

To fill the data gap, we made extensive efforts to collect islanding omics data from multiple sources, normalize them, and integrate them. Based on this data, we constructed the Sperm Maturation Database (SperMD) to provide easy-to-use web services for illustrating multi-dimensional molecular performance relevant to sperm maturation, including data retrieval, visualization, and comparison (Fig. 1). We hope this work could enhance the mechanistic understanding of sperm maturation, support the precise diagnosis of subfertility, and assist in the better application of assisted reproductive techniques (ART).

## Construction and content

### Data acquirement

To establish the database, we keyword-searched the sperm maturation-related articles on PubMed and then derived the related datasets from several public omics data sources such as GEO, E-MTAB, and PRIDE. The matched omics data was manually obtained from the corresponding repositories. Whilst, the experimental parameters were extracted. The terms of reproductive phenotype were mainly downloaded from the Human Phenotype Ontology (HPO, https://hpo.jax.org/app/) and the Mouse Genome Informatics (MGI, https://www.informatics.jax.org/vocab/mp_ontology), respectively. The full list of multi-omics data was given in Supplementary Table 1 of Addictional file1.

### Dataset pre-processing

For 68 bulk RNA-seq transcriptomes, the raw data in FASTQ format were pre-processed through quality control, adapter trimming, and quality filtering using the tool fastp (version 0.20.1, default parameters) [16]. The pre-processed data were further exposed to the salmon tool (version 1.6.0, default parameters) to calculate transcript abundance based on the read counts [17], in which the salmon_sa_index was built by referring to the human GRCh38 primary assembly or the mouse GRCm39 primary assembly, respectively. Both genome references were downloaded from GENCODE (https://www.gencodegenes.org/). To facilitate the comparison between RNA-seq transcriptomes, the transcript abundance was also normalized by adopting the value of Transcripts Per Million (TPM), which can be calculated by (1):

1 $$\begin{aligned} \mathrm {TPM_i} = \frac{\mathrm {N_i}/\mathrm {L_i}}{\sum _{j}^{}\mathrm {N_j}/\mathrm {L_j}} \times 10^{6} \end{aligned}$$

where $N_{i}$ stands for the reads count mapping to the *i*-th gene and $L_{i}$ stands for effective gene length. The effective gene length is defined as the length of the longest transcript of the *i*-th gene.

For all seven single-cell RNA-seq (scRNA-seq) transcriptomes, we followed the same data pre-processing protocols as that of the original publications and adopted the original cell type annotations. All scRNA-seq data with clean raw gene expression matrix (the count matrix) were downloaded from GEO and then processed with the Seurat V3/V4 functions of NormalizeData, FindVariableFeatures, and ScaleData in order [18, 19]. For every normalized scRNA-seq transcriptome, the cells were clustered with either RunUMAP or RunTSNE according to the suggestions of the original articles. The UMAP or tSNE dimension reduction plots were generated with the ggplot2 package [20]. All genes in the transcriptomes were mapped to the Ensembl ID gene symbol for unification.

Of the total 91 proteomes, 66 proteomes were quantitative proteomes with protein expression matrices and the remaining 25 proteomes were qualitatively determined. All expression matrices except the human seminal plasma (6 proteomes) were first pre-processed by excluding the unreliable protein records that had no expression in more than half of the replicates. The pre-processed expression matrices were subsequently normalized by conducting a $Log_{2}(x+1)$ transformation, where x stands for the expression value. For the human seminal plasma proteomes, we used the normalized expression matrices which were quantitated according to the spectral counting. For all proteomes, the protein identifiers were mapped to the UniProt accession number (AC) for unification. The qualitative proteomes (25 proteomes) needed no pre-processing other than coordinating the protein with the UniProt AC.

From three literature sources, we obtained information on 62 distinct metabolites in human spermatozoa and seminal plasma which were differentially expressed between the infertile and fertile samples, satisfying p-value<0.05. The physiochemical particulars of metabolites were derived from the HMDB database.

### Database implementation

SperMD was deployed on the Linux-Model View Controller-JavaScript architecture. The MySQL (version 5.7.25) was used to manage the underlying data storage, access, and maintenance. Efficient and user-friendly interfaces were designed and coded with JavaScript for interactive data retrieval, visualization, and comparison. SperMD can be freely accessed at http://bio-add.org/SperMD.

### Data statistics of SperMD

**Fig. 2:**
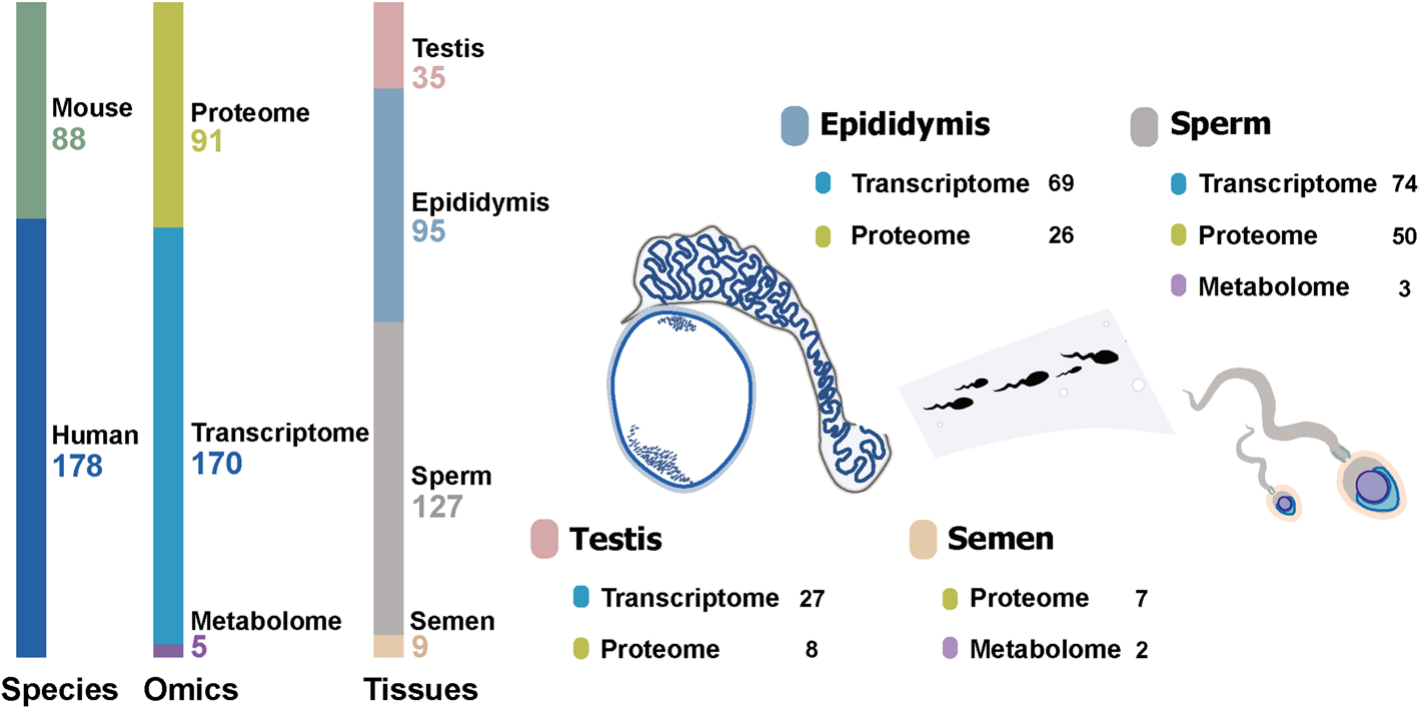
Statistics of SperMD by data types, tissues, and species

SperMD collects 266 distinct sets of multi-omics data from 60 publications and one GEO Series, covering both human (120 transcriptomes, 53 proteomes and five human metabolomes) and mouse (50 transcriptomes and 38 proteomes) (Fig. 2). By omics types, the database incorporates 170 transcriptomes (163 bulk RNA-seq transcriptomes and seven scRNA-seq transcriptomes), 91 proteomes (53 proteomes from human and 38 proteomes from mouse), and five human metabolomes. By tissues, it covers the major tissues related to the development process of sperm maturation, including testis (27 transcriptomes and 8 proteomes), epididymis (69 transcriptomes and 26 proteomes), spermatozoa (74 transcriptomes, 50 proteomes and three metabolomes), and semen (7 proteomes and two metabolomes). By molecules, the proteomes cover 12,156 human proteins and 6,523 mouse proteins; whereas the transcriptomes cover 21,018 human genes and 21,496 mouse genes, more than 93% of these genes are mutual to bulk transcriptomes and single-cell transcriptomes (Supplementary Fig 1 in Additional file 1).

### Database access

**Fig. 3:**
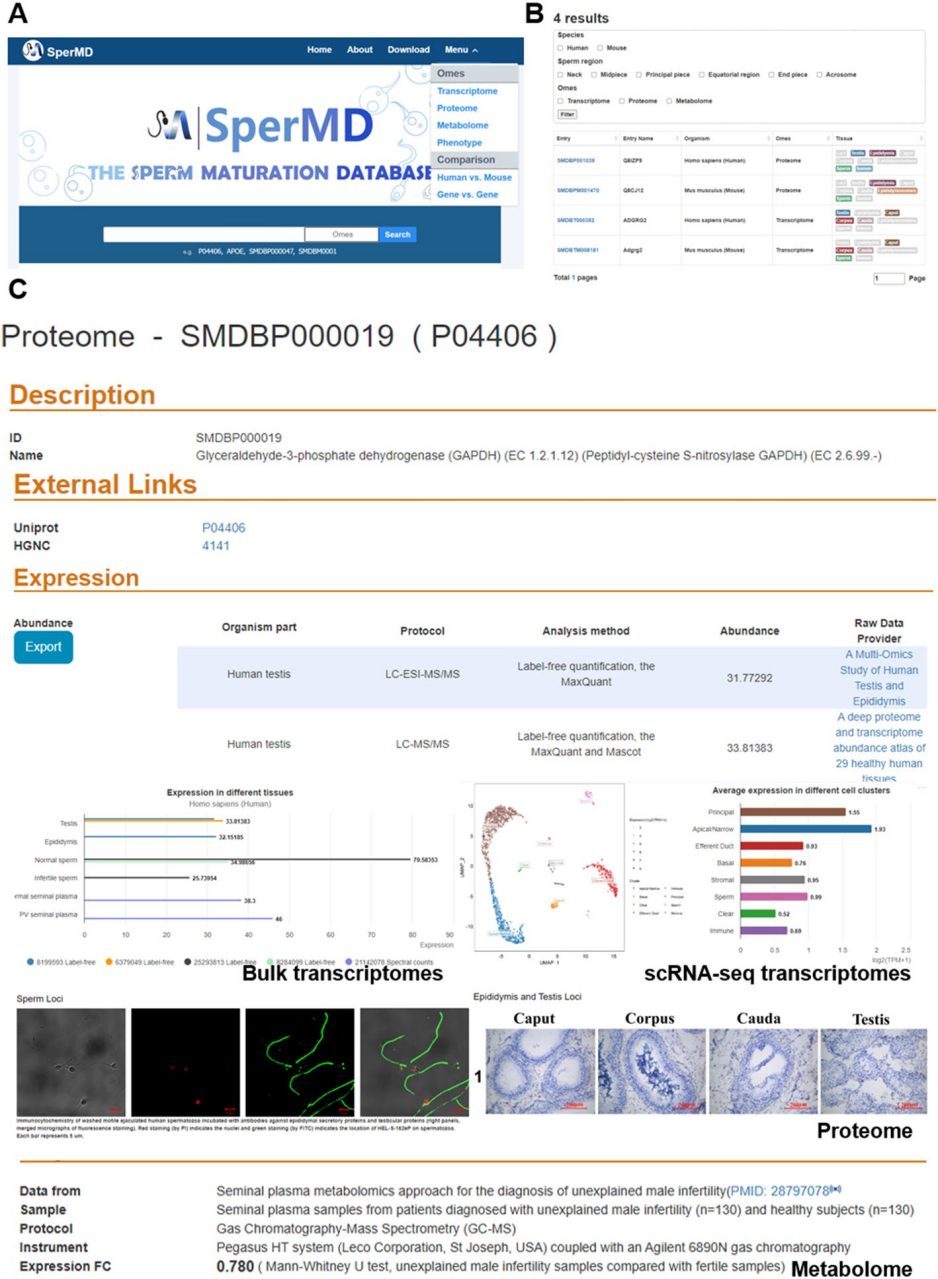
The web services of SperMD.**A** Data search of SperMD. **B** The hits of database search, using keyword â€˜aass’ as the example. **C** Illustration of the Result page

The SperMD database can be freely accessed at http://bio-add.org/SperMD without reregistration. In general, the database supports data retrieval in two ways: Search or Browse (Fig. 3A). The Search method enables a simple keyword search of the database upon the input of the full or partial terms of gene symbols, protein names, metabolite names and phenotype terms. Wild characters such as “*/.” are not allowed. The Search method can be optionally narrowed down to any scope of proteome, transcriptome, metabolome and phenotype. The hits are displayed in a datasheet, sorted by subjects of entry ID, entry name, species, ome type, and tissue (Fig. 3B). In addition to the Search method, the database also provides the Browse method for rapid data retrieval. All gene/protein entries are sorted in alphabetical order by categories of proteome, transcriptome, and metabolome.

The Result presents detailed information on the selected hits in separate pages of transcriptome, proteome, or metabolome. For the definite gene or protein, the pages of transcriptome and proteome are switchable. The Result pages present the information in five sections, including Description, External Links, Expression, Loci, and Biological Properties (Fig. 3C). The Description section and the Biological Properties section describe the molecular particulars and the functional annotations (Gene ontology or KEGG ontology), respectively. The External Links section offers the crosslinks of the gene/protein to several related databases such as GenBank, UniProt, ENCODE, HGNC, and HMDB. The Loci section is only available for proteome, which presents valuable immunohistochemical images to illustrate the protein expression locus in the tissues or sub-tissues of spermatozoa, epididymis, and testis. The Expression section provides the core information of the Result. In the case of the transcriptome, the database lists the normalized TPM and FKPM expression values (RMA value for the microarray dataset) in different tissues in a datasheet, which is also visualized in a bar chart for straightforward comparison. When the scRNA-seq Transcriptomes are available, they are presented separately by experiments. Each experiment dataset is composed of two charts: a UMAP or tSNE dimension reduction plot which illustrates the gene expression level in every cell constituent by clusters, and a bar chart which shows the average gene expression levels in different cell types (cell clusters). In the case of the proteome, the database also presents the protein abundance in a datasheet for data details and a bar chart for straightforward comparison, in the same way as that of the transcriptome. In the case of the metabolome, only a datasheet is given to present the fold change (expression FC) of metabolite between two experimental conditions. Besides, the SperMD database also allows to “Export” the customized datasheets of transcriptomes and proteomes.

### Functions: large-scale comparison of molecular expressions

**Fig. 4:**
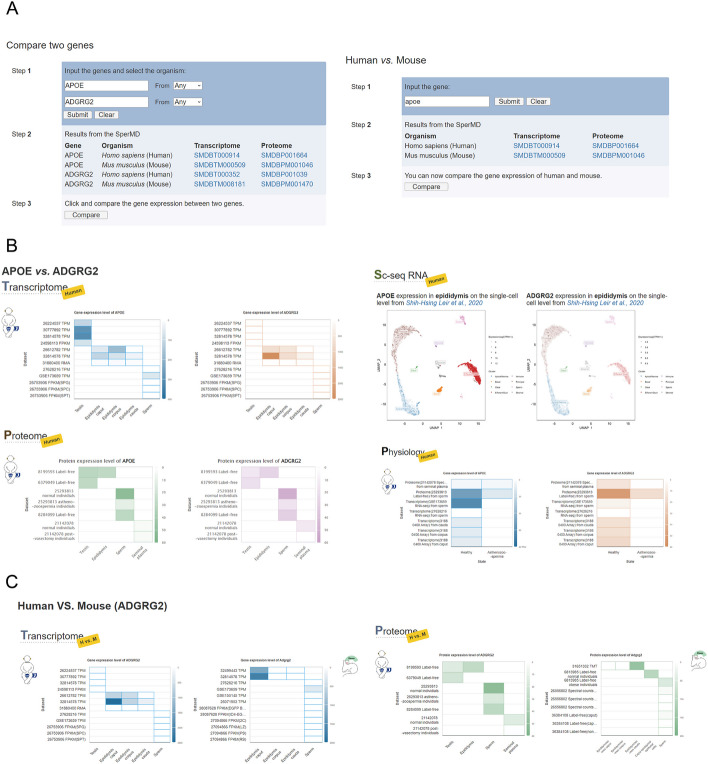
The schematic illustration of gene comparison functions of SperMD. **A** Two implemented tools for large-scale comparison either between genes or between species. **B** Illustration of molecular comparison by omics data types or physiological states. **C** Illustration of molecular comparison between species

Large-scale comparison of gene expression can help reveal gene relationships and review the molecular conservativeness and discrepancies between species. SperMD develops two tools to support direct molecular comparison either between genes or between species. The gene-gene comparison is demonstrated in two back-to-back heat map plots by omes and pathological states if available, one plot for one gene (Fig. 4). The Y-axis of the heat map lists the involved transcriptomes or proteomes, and the X-axis catalogs the involved tissues of the testis, epididymis, epididymis segments (caput, corpus, and cauda), sperm, and seminal plasma when available. Every block in the plot stands for the expression level of the gene or protein, indicated by the colour shade. In the case of the single-cell transcriptome, the gene expression atlas over the cell constituents of the epididymis is illustrated in the unified UMAP or tSNE dimension reduction plots for both genes. Within the plot, each dot stands for a cell and the gene expression level in the cell is indicated by the colour intensity. The expression levels in different pathological states (health vs. disease) are also illustrated in the way of a heat map plot, one plot for one gene. In the heat map plot, the Y-axis lists the transcriptomes or proteomes with the tissue information and the X-axis includes two states of health (health or fertile) and diseases (asthenozoospermia, oligozoospermia, or infertility). When comparing gene/protein expression between human and mouse, the result is illustrated in a similar way as that of the gene-gene comparison, except replacing two genes with the same gene in two species (Fig. 4C).

## Utility and discussion

### Example application: monitoring the gene expression multi-dimensionally to characterize gene functions

**Fig. 5:**
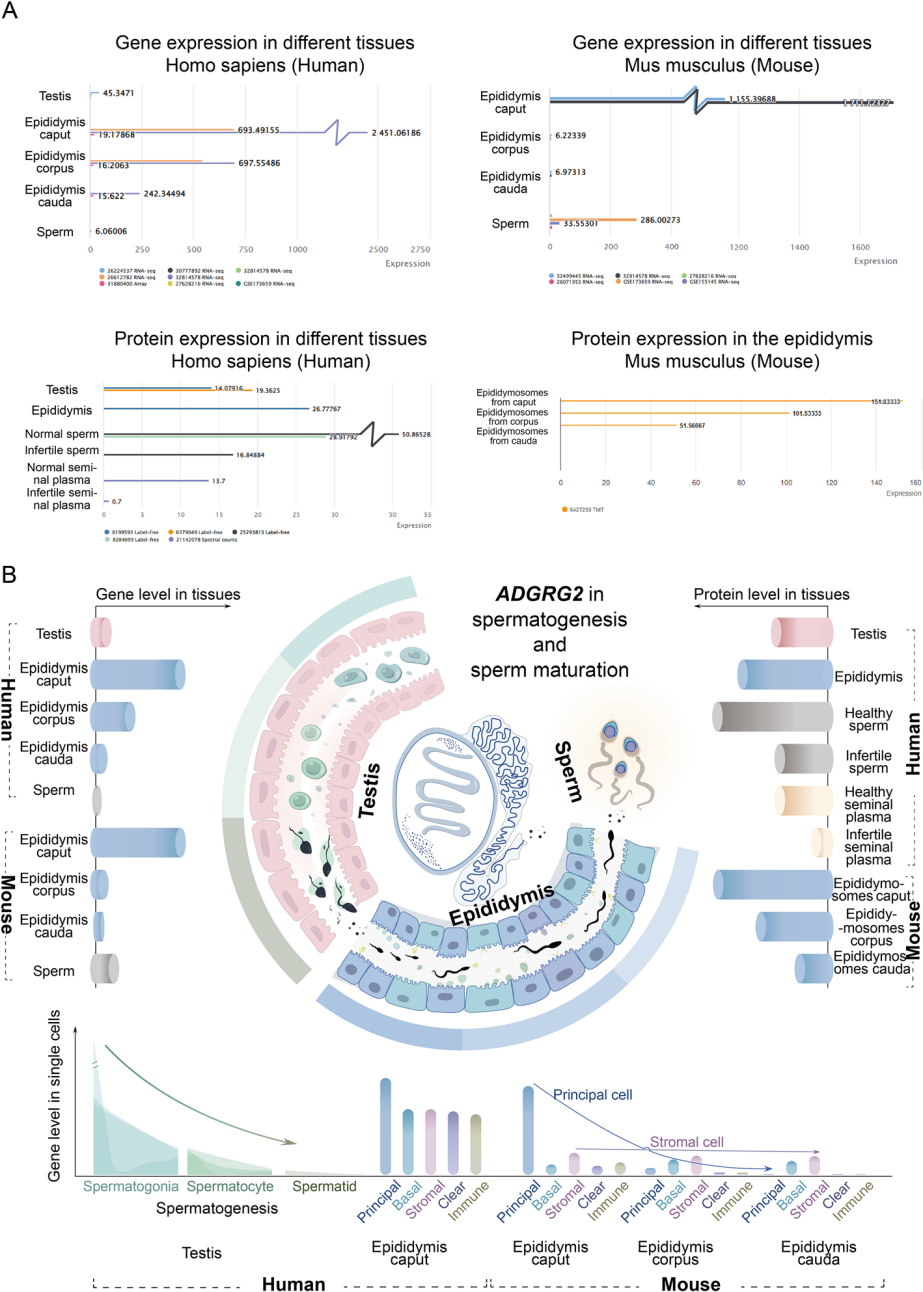
Example application of SperMD in a systematic exploration of ***ADGRG2*** function. **A** Illustration of gene and protein expression profiles of *ADGRG2* over different tissues from the search of SperMD with the keyword “*ADGRG2*”. **B** Integrative illustration of *ADGRG2* expression at the multi-dimensional and multi-granular scales

SperMD provides a one-stop service to monitor gene expression change at multi-dimensional scales. This will be particularly valuable for characterizing gene functions in sperm maturation systematically and further excavating gene potential as the therapeutic targets in countermining male infertility or subfertility. Here, we used *ADGRG2* as an example to illustrate how to explore gene functions systematically, empowered by a search of SperMD (Fig. 5). *ADGRG2* (adhesion G-protein-coupled receptor G2) belongs to the G-protein-coupled receptor (GPCR) family. Many pieces of evidence suggest that *ADGRG2* may participate in the male reproductive duration [21, 22]; regretfully, the exact roles haven’t been fully illuminated yet.

Search against SperMD manifested that the *ADGRG2* gene ubiquitously expresses in the testis, epididymis, sperm, and semen (Fig. 5A). However, compared to the testis and spermatozoa, the gene expression is extremely abundant in the epididymis (in particular at the caput segment), suggesting the potential active roles of *ADGRG2* in the epididymis caput. This speculation is consolidated by the consistently high expression of *ADGRG2* protein in human and mouse epididymis caput. However, the exact role of *ADGRG2* still remains unclear. Previously, knockout of *ADGRG2* in mice was reported to decrease sperm number, flagella abnormality, dysregulation of fluid reabsorption, and sperm accumulation in the efferent ducts at the junction of the testis and epididymis [23]. The *ADGRG2* knockout could decrease the expression of approximately 30 epididymis-specific genes in the epididymis caput of mice [24], including *CRES* (*CST8*) which was found in extracellular vesicles of the epididymis and likely participated in the delivery of maturation-associated molecules [25]. Another work reported that, compared to the non-obstructive azoospermia patients, the azoospermia patients with *ADGRG2* mutant almost didn’t express *ADGRG2* in the proximal epididymis [26]. Hence, it is reasonable to infer the high expression of *ADGRG2* is required to maintain the epididymis function for male fertility.

Looking closely into the epididymis, *ADGRG2* highly selectively expresses in the principal cells of epididymis caput and is dramatically down-regulated from caput to corpus to cauda (Fig. 5B); the same expression pattern is also observed in mice. These results cement that the epididymis caput could be the place where *ADGRG2* functions. Together with the previous finding on *ADGRG2* function in re-absorbing the fluid from the testis or efferent tubules [27], it can be inferred that the high expression of *ADGRG2* in the principal cells of the epididymis caput likely plays a predominant endocytotic role in maintaining the epididymis structure and epididymal microenvironment for sperm maturation by re-absorbing the tubular fluid.

Noteworthy, the *Adgrg2* protein highly expresses in healthy sperm, about three times more than that in the infertile sperm (Fig. 5A), suggesting *Adgrg2* is also likely associated with spermatogenesis. To explore this, we integrated the scRNA-seq data from three independent experiments in SperMD and plotted the *ADGRG2* expression levels in different cell stages of three successive development phases (spermatogonia, spermatocyte, and spermatid) to achieve a zoom-in view (Fig. 5B). *ADGRG2* selectively expresses in the spermatogonial stem cells (SSCs) and then gradually reduces its expression to almost null throughout spermatogenesis. This finding suggests that *ADGRG2* may serve as the starting signal or inhibiting factor to spermatogenesis. Collectively, by reviewing the multi-dimensional and multi-granular expression data, it can infer the multiple gene functions of *ADGRG2* in both spermatogenesis and sperm maturation with substantial data support. This provides clear clues for future experimental validation.

### Discussion

**Table 1:** Comparison of SperMD with several sperm-related databases

**Name**	**Species**	**Tissue types**	**Data type**	**Data set number**	**Last update**	**Availability**	**URL**
		Testis	Transcriptome				
		Epididymis	Proteome				
		Sperm	Metabolome				
**SperMD**	Human and mouse	Semen		266	**2022**.**10**	$\checkmark$	http://bio-add.org/SperMD
		Testis, Epididymis					
The Mammalian Reproductive Genetics Database V2	Human, mouse and rat	Sperm, Female reproductive and non-reproductive tissues and cells	Transcriptome	988	2022.05	$\checkmark$	https://orit.research.bcm.edu/MRGDv2
		Sperm					
SpermBase	Human, mouse, rat, rabbit	Sperm head	Transcriptome	32	2016.11	$\checkmark$	http://spermbase.org/
		Testis					
REPROD-UCTION-2DPAGE	Human and mouse	Sperm	Proteome	8	2013.07	$\checkmark$	http://reprod.njmu.edu.cn/cgi-bin/2d/2d.cg
SpermTree	4,705 unique species	Sperm	Morphological descriptions of sperm morphology	–	2022.01	$\checkmark$	https://spermtree.org/
		Testis					
		Sperm					
The Male Fertility Gene Atlas	Human	Brain etc.	Overview of research results of different omes	115	2020.09	$\checkmark$	https://mfga.uni-muenster.de/projectInfo.html
HSPD	Human	Sperm	Proteome	1	2012.10	$\times$	–
HTPD	Human	Testis	Proteome	1	2012.10	$\times$	–
Proteome database of human seminal plasma	Human	Seminal plasma	Proteome	1	2006.05	$\times$	–

Since the beginning of this century, the power of omics technologies in sperm research has been recognized [28, 29]. Thereafter, hundreds of omics experiments were conducted for different biological targets, providing new insights into the field of male reproductive health. The recent emergence of cutting-edge scRNA-seq technology further promotes research into a new realm by providing the unprecedented resolution of individual cell expression changes of human and mouse epididymis [30–32]. Doubtlessly, twenty years of omics endeavours have extensively enriched our knowledge of sperm maturation. Regretfully, until this study, few actions have ever been made to collect, normalize, and integrate the heterogeneous omics data to revisit the molecular performance during sperm maturation. In this study, we make an audacious attempt at data integration whereby depict the multi-dimensional (both spatially and temporally), multi-granular (tissue, segment, and cell), and multi-contextual (gene, protein, and metabolite) portrayal of molecular expression landscapes for sperm maturation in both humans and mice. According to the open literature, similar work has not been reported previously. Here, we make an additional comparison of SperMD with several sperm-related databases and summarize the results in Table 1. Collectively, the newly constructed SperMD could serve as a valuable data resource for aiding the systematic exploration of sperm maturation.

SperMD provides a broad scene of molecular performance to solve the ambiguity caused by the data islanding. For instance, we combine multiple transcriptomes and proteomes to consolidate whether the genes consistently transcribe and translate with high abundance under a spatiotemporal condition. Besides, the functions of SperMD enable thorough molecular comparison between genes or between human and mouse at multiple scales. This endeavor will help recognize the functional gap caused by the species divergence and link genes together under the big picture of sperm maturation.

To keep pace with the rapidly developed field of male fertility, we are scheduled to update the database annually by developing new functions and incorporating new contents; however, minor modifications of the database such as bug-fixings and adding new data will be undertaken when applicable.

## Conclusion

In summary, the SperMD database provides a multi-dimensional molecular performance in various scenarios of sperm maturation. It broadens the mechanistic investigation of sperm maturation extensively to multiple spatiotemporal dimensions. It will also prompt accurate diagnosis and enlighten precise therapeutic regimens of male subfertility in clinical practices.

### Supplementary Information

**Additional file 1.** Statistics analysis and data sources information of SperMD.

## Data Availability

The experiment conditions and literature PMIDs of all omics datasets were given in Supplementary Table 1 of Addictional file 1. The raw data can be acquired from public repositories according to the literature descriptions. The comprehensive expression information of genes/proteins/metabolites can be retrieved from the SperMD database at http://bio-add.org/SperMD.
