# Supplementary material for: SperMD: the expression atlas of sperm maturation
Source: BMC Bioinformatics. 2024 Jan 17;25:29. doi: 10.1186/s12859-024-05631-x (PMC10792849; doi:10.1186/s12859-024-05631-x)
Supplement: Supplementary file 1 — Additional file 1. Statistics analysis and data sources information of SperMD. [file 12859_2024_5631_MOESM1_ESM.docx]

**Supplementary Information**

**Statistics analysis and data sources information of SperMD.**

Yifan Li^1^, Qianying Li^2^, Lvying Wu^2^, Haiyan Wang^3^, Hui Shi^4^, Chenhui Yang^1^, Yiqun Gu^5,*^, Jianyuan Li^3,5,*^ and Zhi-Liang Ji^2,*^

^1^ School of Informatics, National Institute for Data Science in Health and Medicine, Xiamen University, Xiamen, Fujian 361102, China

^2^ State Key Laboratory of Cellular Stress Biology, School of Life Sciences, Faculty of Medicine and Life Sciences, Xiamen University, Xiamen, Fujian 361102, China

^3^ Shandong Epihealth Biotech Ltd., Yantai, Shandong 264670, PR China

^4^ College of Life Science, Yantai University, Yantai, Shandong 264005, PR China

^5^ Institute of science and technology, National Health Commission, Beijing 100081, PR China

* To whom correspondence should be addressed. Email: Zhiliang Ji([appo@xmu.edu.cn](mailto:appo@xmu.edu.cn)); Tel: +86 0592 2182897; fax: +86 0592 2182897.


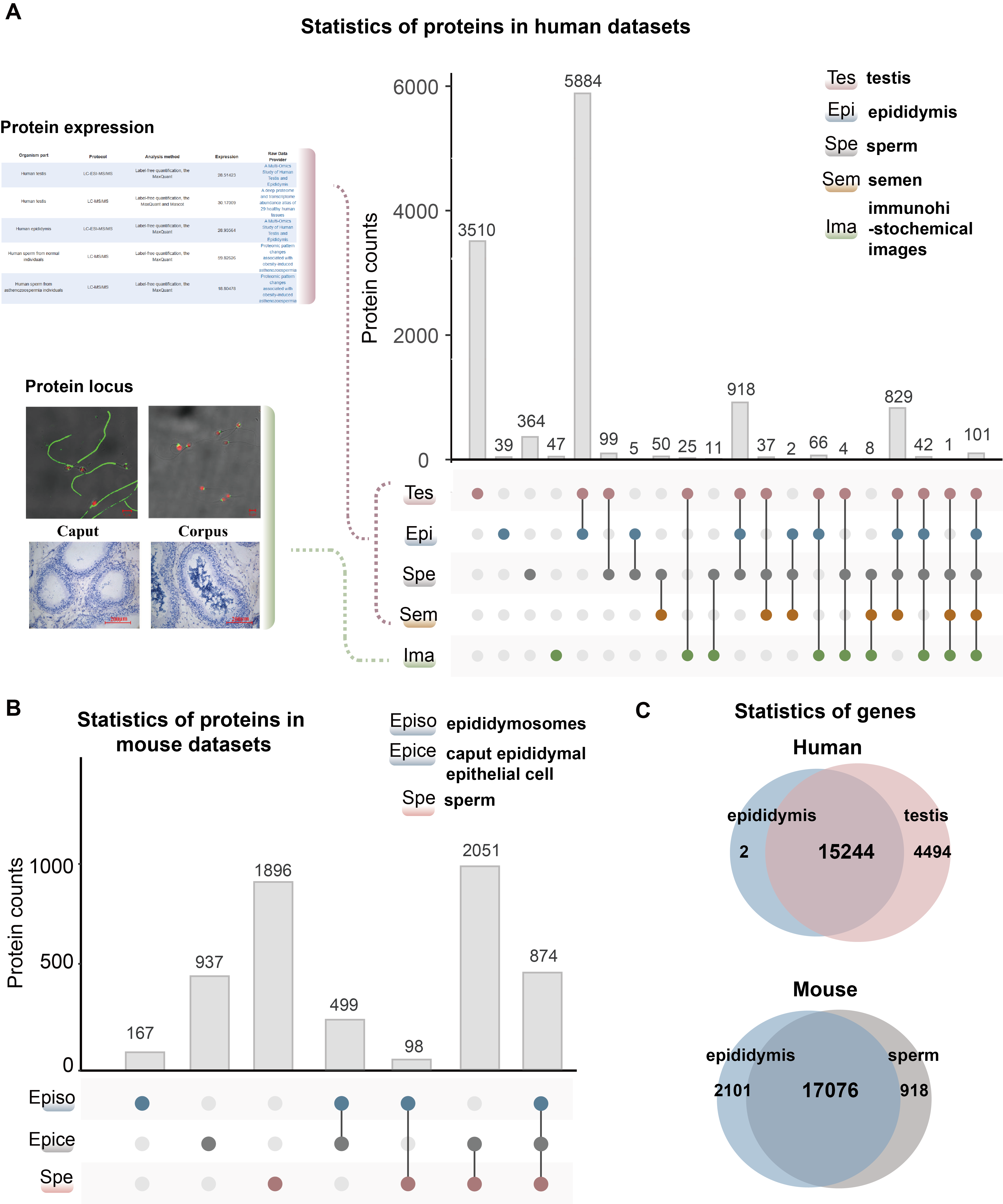


**Supplementary Fig 1.** The statistics of expression at different levels. (**A**) The statistics of protein expression in human. In this section, immunohistochemical images located in sperm and epididymis tissue are displayed details. (**B**) The statistics of protein expression in mouse. (**C**) The statistics of gene expression at single-cell level in human and mouse.

**Supplementary Table 1. Human and mouse data sources**

Proteome of human

| **Sample** | **Proteome** | **Age** | **PMID** | **Protocol** |
| --- | --- | --- | --- | --- |
| **Testis** | 1 | 37 years old | PMID:23436708[1] | 2D HPLC-MS/MS |
| **Testis** | 1 | 27-32 years old | PMID:21178120[2] | MALDI-TOF MS/MS |
| **Testis** | 1 | 35 years old | PMID:21136815[3] | RP-LC-MS/MS |
| **Testis** | 1 | 56 years old | PMID: 30777892[4] | LC-MS/MS |
| **Testis and caput, corpus and cauda of epididymis** | 16 | Adult | PMID:34199411[5] | LC-MS/MS |
| **Epididymis** | 1 | 27-32 years old | PMID:20736409[6] | MALDI-TOF MS |
| **Normal sperm** | 1 | Adult | PMID:16819732[7] | MALDI-TOF MS |
| **Normal sperm** | 1 | Adult | PMID:21136703[8] | LC-MS/MS |
| **Sperms from infertile patients** | 1 | Adult | PMID:18040982[9] | MALDI-TOF MS |
| **Normal sperm** | 1 | 21.38 ± 2.39 years old | PMID:17486272[10] | MALDI-TOF/TOF MS |
| **Normal sperm** | 1 | 30 ± 4years old | PMID:23268119[11] | LC–MS/MS |
| **Normal sperm** | 1 | Adult | PMID:20078857[12] | LC-MS and Edman degradation |
| **Normal sperm surface** | 1 | Adult | PMID:19962198[13] | LC-MS and Edman degradation |
| **Normal sperm nuclei** | 1 | Adult | PMID:21630459[14] | LC-MS/MS |
| **Normal sperm surface** | 1 | 22-32 years old | PMID:21559292[15] | LC-MS/MS |
| **Normal sperm** | 1 | Adult | PMID:21792924[16] | LC-MS/MS |
| **Normal sperm tail** | 1 | Adult | PMID:23161514[17] | LC-MS/MS |
|  |  |  |  |  |
| **Sample** | **Proteome** | **Age** | **PMID** | **Protocol** |
| **Normal sperm head and flagella** | 1 | Adult | PMID:23161668[18] | LC-MS/MS |
| **Normal and asthenozoospermic sperm** | 1 | Adult | PMID:18281682[19] | LC-MS/MS |
| **Normal and asthenozoospermic sperm** | 1 | Adult | PMID:20304782[20] | MALDI MS/MS |
| **Normal sperm** | 1 | Adult | PMID:23893156[21] | 2D nanoUPLC-ESI-MS |
| **Normal and asthenozoospermic sperm** | 1 | 20-40 years old | PMID:27895139[22] | UPLC-MS |
| **Normal and asthenozoospermic sperm** | 1 | Adult | PMID:25250979[23] | LC-MS/MS |
| **Normal sperm** | 1 | Adult | PMID:24781426[24] | LC-MS/MS |
| **Normal and asthenozoospermic (obesity-associated) sperm** | 6 | Adult | PMID:25293813[25] | LC-MS/MS |
| **Normal sperm** | 1 | Adult | PMID:34277615[26] | LC-MS/MS |
| **Seminal plasma** | 1 | Adult | PMID:25927022[27] | 2-DE, SDS-PAGE-LC-MS/MS, MudPIT |
| **Seminal plasma from fertile people and post-vasectomy patients** | 6 | Adult | PMID:21142078[28] | Spectral counting |

Transcriptome of human

| **Sample** | **Transcriptome** | **Age** | **PMID** | **Protocol** |
| --- | --- | --- | --- | --- |
| **Testis** | 8 | Adult | PMID:30777892[4] | RNA-seq |
| **Testis** | 2 | Adult | PMID:26224537[29] | RNA-seq |
| **Testis** | 8 | 26-62 years old | PMID:24598113[30] | RNA-seq |
| **Testis, caput, corpus and cauda of epididymis** | 12 | 40,52,65 years old | PMID:32814578[31] | RNA-seq |
| **Sample** | **Transcriptome** | **Age** | **PMID** | **Protocol** |
| **Epididymis and primary cultures from epithelial cells in caput, corpus and cauda** | 15 | 22–36 years old | PMID:26612782[32] | RNA-seq |
| **Caput, corpus and cauda of epididymis** | 24 | 37,50,52 years old | PMID:31880400[33] | Chip array |
| **Human adult spermatogonial stem cells** | 18 | Adult | PMID:28985528[34] | RNA-seq |
| **Sperm with normal spermatogenesis** | 9 | 21-45 years old | PMID:26753906[35] | RNA-seq |
| **Normal sperm** | 6 | 18-55 years old | PMID:34174366[36] | RNA-seq |
| **Normal sperm** | 3 | Adult | PMID:27628216[37] | RNA-seq |
| **Testis and normal sperm** | 11 | Adult | PMID:23471003[38] | RNA-seq |
| **Testis** | 1 | 17, 24, 25 years old | PMID:30315278[39] | Single-cell RNA-seq |
| **Testis** | 1 | 7, 11, 13 and 14 years old | PMID:31928944[40] | Single-cell RNA-seq |
| **Caput epithelial of epididymis** | 1 | 24 (2), 31, 32, 38, 47, 57 years old | PMID:32855272[41] | Single-cell RNA-seq |
| **Testis from profile people, OA (obstructive azoospermia) and NOA (nonobstructive azoospermia)** | 1 | 30, 60 years old (Normal); 39, 43, 27, 34, 44, 41, 29 years old (OA); 24 years old (NOA) | PMID:30174296[42] | Single-cell RNA-seq |

Metabolome of human

| **Sample** | **Metabolome** | **Age** | **PMID** | **Protocol** |
| --- | --- | --- | --- | --- |
| **Normal and asthenozoospermic sperm** | 2 | 21-32 years old | PMID:29682560[43] | GC-MS |
| **Normal and asthenozoospermic sperm** | 1 | Adult | PMID:25854681[44] | GC-MS |
| **Sperm from patients with unexplained male infertility (UMI)** | 2 | Adult | PMID:28797078[45] | GC-MS |

Proteome of mouse

| **Sample** | **Proteome** | **Age** | **PMID** | **Protocol** |
| --- | --- | --- | --- | --- |
| **Epididymis** | 1 | Adult | PMID:26225126[46] | MALDI-TOF-MS |
| **Sperm acrosomal matrix** | 1 | Adult | PMID: 22707618[47] | LC-MS/MS |
| **Epididymosomes from caput, corpus and cauda of epididymis** | 9 | Adult | PMID:30213844[48] | LC-MS/MS |
| **Sperm from normal and fat mouse** | 12 | 14 weeks | PMID:31651332[49] | LC-MS/MS |
| **Sperm from the caput, corpus and cauda of epididymis**  **Caput epithelial of epididymis** | 3 | Adult  Adult (8–12 weeks) | PMID: 26556802[50] | LC-MS/MS  nanoLC-MS/MS |
|  |  |  |  |  |
|  |  |  |  |  |
| **Sample** | **Proteome** | **Age** | **PMID** | **Protocol** |
| **Sperm from caput, non-capacitated and capacitated sperm from cauda** | 9 | Adult (8 weeks) | PMID: 35392627[51] | nLC-MS/MS |
|  |  |  |  |  |
| **Epididymis** | 1 | Adult | PMID: 36384108[52] | MALDI-TOF-MS |

Transcriptome of mouse

| **Sample** | **Transcriptome** | **Age** | **PMID** | **Protocol** |
| --- | --- | --- | --- | --- |
| **Caput, corpus and cauda of epididymis** | 9 | Adult | PMID:32814578[31] | RNA-seq |
| **Caput, corpus and cauda of epididymis** | 9 | Adult | PMID:32499443[53] | RNA-seq |
| **whole mouse sperm and fractionated nuclei** | 6 | Adult | PMID:26071953[54] | RNA-seq |
| **Normal sperm** | 5 | Adult | PMID:34174366[36] | RNA-seq |
| **ID4-EGFP^Bright^and ID4-EGFP^Dim^spermatogonia** | 6 | Adult | PMID:28087628[55] | RNA-seq |
| **Sample** | **Transcriptome** | **Age** | **PMID** | **Protocol** |
| **Purified spermatogenic stage-specific cells** | 4 | 10–11and 24–25dpp | PMID:27094866[56] | RNA-seq |
| **Normal and nicotine-treated sperm** | 4 | Adult | GSE155145[57](unpublished) | RNA-seq |
| **Normal sperm and normal sperm head** | 4 | Adult | PMID:27628216[37] | RNA-seq |
| **Spermatogenic cells** | 1 | 6–8 weeks | PMID:30061742[58] | sc-RNA seq |
| **Caput, corpus, cauda and vas deferens of epididymis** | 1 | 10-12 weeks | PMID:32729827[59] | sc-RNA seq |
| **Caput, corpus and cauda of epididymis** | 1 | 42 days and 56 days | PMID:34001862[60] | sc-RNA seq |

**References**

1. Liu M, Hu Z, Qi L, Wang J, Zhou T, Guo Y, et al. Scanning of novel cancer/testis proteins by human testis proteomic analysis. Proteomics. 2013;13:1200–10.

2. Li J, Liu F, Liu X, Liu J, Zhu P, Wan F, et al. Mapping of the human testicular proteome and its relationship with that of the epididymis and spermatozoa. Mol Cell Proteomics. 2011;10:M110.004630.

3. X G, P Z, R H, Z Z, J S. Analysis of the human testis proteome by mass spectrometry and bioinformatics. Proteomics Clinical applications. 2008;2.

4. Wang D, Eraslan B, Wieland T, Hallström B, Hopf T, Zolg DP, et al. A deep proteome and transcriptome abundance atlas of 29 healthy human tissues. Mol Syst Biol. 2019;15:e8503.

5. Zheng W, Zhang Y, Sun C, Ge S, Tan Y, Shen H, et al. A Multi-Omics Study of Human Testis and Epididymis. Molecules. 2021;26:3345.

6. Li J, Liu F, Wang H, Liu X, Liu J, Li N, et al. Systematic mapping and functional analysis of a family of human epididymal secretory sperm-located proteins. Mol Cell Proteomics. 2010;9:2517–28.

7. Martínez-Heredia J, Estanyol JM, Ballescà JL, Oliva R. Proteomic identification of human sperm proteins. Proteomics. 2006;6:4356–69.

8. Baker MA, Reeves G, Hetherington L, Müller J, Baur I, Aitken RJ. Identification of gene products present in Triton X-100 soluble and insoluble fractions of human spermatozoa lysates using LC-MS/MS analysis. Proteomics Clin Appl. 2007;1:524–32.

9. de Mateo S, Martínez-Heredia J, Estanyol JM, Domínguez-Fandos D, Domíguez-Fandos D, Vidal-Taboada JM, et al. Marked correlations in protein expression identified by proteomic analysis of human spermatozoa. Proteomics. 2007;7:4264–77.

10. Li L-W, Fan L-Q, Zhu W-B, Nien H-C, Sun B-L, Luo K-L, et al. Establishment of a high-resolution 2-D reference map of human spermatozoal proteins from 12 fertile sperm-bank donors. Asian J Androl. 2007;9:321–9.

11. Wang G, Guo Y, Zhou T, Shi X, Yu J, Yang Y, et al. In-depth proteomic analysis of the human sperm reveals complex protein compositions. J Proteomics. 2013;79:114–22.

12. Naaby-Hansen S, Diekman A, Shetty J, Flickinger CJ, Westbrook A, Herr JC. Identification of calcium-binding proteins associated with the human sperm plasma membrane. Reprod Biol Endocrinol. 2010;8:6.

13. Naaby-Hansen S, Herr JC. Heat shock proteins on the human sperm surface. J Reprod Immunol. 2010;84:32–40.

14. de Mateo S, Castillo J, Estanyol JM, Ballescà JL, Oliva R. Proteomic characterization of the human sperm nucleus. Proteomics. 2011;11:2714–26.

15. Gu B, Zhang J, Wu Y, Zhang X, Tan Z, Lin Y, et al. Proteomic analyses reveal common promiscuous patterns of cell surface proteins on human embryonic stem cells and sperms. PLoS One. 2011;6:e19386.

16. Nixon B, Mitchell LA, Anderson AL, McLaughlin EA, O’bryan MK, Aitken RJ. Proteomic and functional analysis of human sperm detergent resistant membranes. J Cell Physiol. 2011;226:2651–65.

17. Amaral A, Castillo J, Estanyol JM, Ballescà JL, Ramalho-Santos J, Oliva R. Human sperm tail proteome suggests new endogenous metabolic pathways. Mol Cell Proteomics. 2013;12:330–42.

18. Baker MA, Naumovski N, Hetherington L, Weinberg A, Velkov T, Aitken RJ. Head and flagella subcompartmental proteomic analysis of human spermatozoa. Proteomics. 2013;13:61–74.

19. Martínez-Heredia J, de Mateo S, Vidal-Taboada JM, Ballescà JL, Oliva R. Identification of proteomic differences in asthenozoospermic sperm samples. Hum Reprod. 2008;23:783–91.

20. Siva AB, Kameshwari DB, Singh V, Pavani K, Sundaram CS, Rangaraj N, et al. Proteomics-based study on asthenozoospermia: differential expression of proteasome alpha complex. Mol Hum Reprod. 2010;16:452–62.

21. Intasqui P, Camargo M, Del Giudice PT, Spaine DM, Carvalho VM, Cardozo KHM, et al. Unraveling the sperm proteome and post-genomic pathways associated with sperm nuclear DNA fragmentation. J Assist Reprod Genet. 2013;30:1187–202.

22. Saraswat M, Joenväärä S, Jain T, Tomar AK, Sinha A, Singh S, et al. Human Spermatozoa Quantitative Proteomic Signature Classifies Normo- and Asthenozoospermia. Mol Cell Proteomics. 2017;16:57–72.

23. Amaral A, Paiva C, Attardo Parrinello C, Estanyol JM, Ballescà JL, Ramalho-Santos J, et al. Identification of proteins involved in human sperm motility using high-throughput differential proteomics. J Proteome Res. 2014;13:5670–84.

24. Azpiazu R, Amaral A, Castillo J, Estanyol JM, Guimerà M, Ballescà JL, et al. High-throughput sperm differential proteomics suggests that epigenetic alterations contribute to failed assisted reproduction. Hum Reprod. 2014;29:1225–37.

25. Liu Y, Guo Y, Song N, Fan Y, Li K, Teng X, et al. Proteomic pattern changes associated with obesity-induced asthenozoospermia. Andrology. 2015;3:247–59.

26. Kumar P, Wang M, Isachenko E, Rahimi G, Mallmann P, Wang W, et al. Unraveling Subcellular and Ultrastructural Changes During Vitrification of Human Spermatozoa: Effect of a Mitochondria-Targeted Antioxidant and a Permeable Cryoprotectant. Front Cell Dev Biol. 2021;9:672862.

27. Gilany K, Minai-Tehrani A, Savadi-Shiraz E, Rezadoost H, Lakpour N. Exploring the human seminal plasma proteome: an unexplored gold mine of biomarker for male infertility and male reproduction disorder. J Reprod Infertil. 2015;16:61–71.

28. Batruch I, Lecker I, Kagedan D, Smith CR, Mullen BJ, Grober E, et al. Proteomic analysis of seminal plasma from normal volunteers and post-vasectomy patients identifies over 2000 proteins and candidate biomarkers of the urogenital system. J Proteome Res. 2011;10:941–53.

29. Jodar M, Sendler E, Krawetz SA. The protein and transcript profiles of human semen. Cell Tissue Res. 2016;363:85–96.

30. Djureinovic D, Fagerberg L, Hallström B, Danielsson A, Lindskog C, Uhlén M, et al. The human testis-specific proteome defined by transcriptomics and antibody-based profiling. Mol Hum Reprod. 2014;20:476–88.

31. Robertson MJ, Kent K, Tharp N, Nozawa K, Dean L, Mathew M, et al. Large-scale discovery of male reproductive tract-specific genes through analysis of RNA-seq datasets. BMC Biol. 2020;18:103.

32. Browne JA, Yang R, Leir S-H, Eggener SE, Harris A. Expression profiles of human epididymis epithelial cells reveal the functional diversity of caput, corpus and cauda regions. Mol Hum Reprod. 2016;22:69–82.

33. Légaré C, Sullivan R. Differential gene expression profiles of human efferent ducts and proximal epididymis. Andrology. 2020;8:625–36.

34. Guo J, Grow EJ, Yi C, Mlcochova H, Maher GJ, Lindskog C, et al. Chromatin and Single-Cell RNA-Seq Profiling Reveal Dynamic Signaling and Metabolic Transitions during Human Spermatogonial Stem Cell Development. Cell Stem Cell. 2017;21:533-546.e6.

35. Zhu Z, Li C, Yang S, Tian R, Wang J, Yuan Q, et al. Dynamics of the Transcriptome during Human Spermatogenesis: Predicting the Potential Key Genes Regulating Male Gametes Generation. Sci Rep. 2016;6:19069.

36. Bianchi E, Stermer A, Nolan T, Li H, Hall S, Boekelheide K, et al. Highly conserved sperm function-related transcripts across three species: human, rat and mouse. Reprod Toxicol. 2021;104:44–51.

37. Schuster A, Tang C, Xie Y, Ortogero N, Yuan S, Yan W. SpermBase: A Database for Sperm-Borne RNA Contents. Biol Reprod. 2016;95:99.

38. Sendler E, Johnson GD, Mao S, Goodrich RJ, Diamond MP, Hauser R, et al. Stability, delivery and functions of human sperm RNAs at fertilization. Nucleic Acids Res. 2013;41:4104–17.

39. Guo J, Grow EJ, Mlcochova H, Maher GJ, Lindskog C, Nie X, et al. The adult human testis transcriptional cell atlas. Cell Res. 2018;28:1141–57.

40. Guo J, Nie X, Giebler M, Mlcochova H, Wang Y, Grow EJ, et al. The Dynamic Transcriptional Cell Atlas of Testis Development during Human Puberty. Cell Stem Cell. 2020;26:262-276.e4.

41. Leir S-H, Yin S, Kerschner JL, Cosme W, Harris A. An atlas of human proximal epididymis reveals cell-specific functions and distinct roles for CFTR. Life Sci Alliance. 2020;3:e202000744.

42. Wang M, Liu X, Chang G, Chen Y, An G, Yan L, et al. Single-Cell RNA Sequencing Analysis Reveals Sequential Cell Fate Transition during Human Spermatogenesis. Cell Stem Cell. 2018;23:599-614.e4.

43. Zhao K, Zhang J, Xu Z, Xu Y, Xu A, Chen W, et al. Metabolomic Profiling of Human Spermatozoa in Idiopathic Asthenozoospermia Patients Using Gas Chromatography-Mass Spectrometry. Biomed Res Int. 2018;2018:8327506.

44. Paiva C, Amaral A, Rodriguez M, Canyellas N, Correig X, Ballescà JL, et al. Identification of endogenous metabolites in human sperm cells using proton nuclear magnetic resonance ((1) H-NMR) spectroscopy and gas chromatography-mass spectrometry (GC-MS). Andrology. 2015;3:496–505.

45. Qiao S, Wu W, Chen M, Tang Q, Xia Y, Jia W, et al. Seminal plasma metabolomics approach for the diagnosis of unexplained male infertility. PLoS One. 2017;12:e0181115.

46. Liu X, Liu F-J, Jin S-H, Shen X-F, Wang Y-W. In-depth Proteomic mapping of mouse (Mus musculus) epididymal constructive basis for sperm maturation. Proteome Sci. 2015;13:20.

47. Guyonnet B, Zabet-Moghaddam M, SanFrancisco S, Cornwall GA. Isolation and proteomic characterization of the mouse sperm acrosomal matrix. Mol Cell Proteomics. 2012;11:758–74.

48. Nixon B, De Iuliis GN, Hart HM, Zhou W, Mathe A, Bernstein IR, et al. Proteomic Profiling of Mouse Epididymosomes Reveals their Contributions to Post-testicular Sperm Maturation. Mol Cell Proteomics. 2019;18 Suppl 1:S91–108.

49. Peng Y, Zhao W, Qu F, Jing J, Hu Y, Liu Y, et al. Proteomic alterations underlie an association with teratozoospermia in obese mice sperm. Reprod Biol Endocrinol. 2019;17:82.

50. Skerget S, Rosenow MA, Petritis K, Karr TL. Sperm Proteome Maturation in the Mouse Epididymis. PLoS One. 2015;10:e0140650.

51. Trigg NA, Skerrett-Byrne DA, Martin JH, De Iuliis GN, Dun MD, Roman SD, et al. Quantitative proteomic dataset of mouse caput epididymal epithelial cells exposed to acrylamide in vivo. Data Brief. 2022;42:108032.

52. Skerrett-Byrne DA, Anderson AL, Bromfield EG, Bernstein IR, Mulhall JE, Schjenken JE, et al. Global profiling of the proteomic changes associated with the post-testicular maturation of mouse spermatozoa. Cell Rep. 2022;41:111655.

53. Kiyozumi D, Noda T, Yamaguchi R, Tobita T, Matsumura T, Shimada K, et al. NELL2-mediated lumicrine signaling through OVCH2 is required for male fertility. Science. 2020;368:1132–5.

54. Johnson GD, Mackie P, Jodar M, Moskovtsev S, Krawetz SA. Chromatin and extracellular vesicle associated sperm RNAs. Nucleic Acids Res. 2015;43:6847–59.

55. ID4 levels dictate the stem cell state in mouse spermatogonia | Development | The Company of Biologists. https://journals.biologists.com/dev/article/144/4/624/48310/ID4-levels-dictate-the-stem-cell-state-in-mouse. Accessed 14 Nov 2023.

56. da Cruz I, Rodríguez-Casuriaga R, Santiñaque FF, Farías J, Curti G, Capoano CA, et al. Transcriptome analysis of highly purified mouse spermatogenic cell populations: gene expression signatures switch from meiotic-to postmeiotic-related processes at pachytene stage. BMC Genomics. 2016;17:294.

57. Qiao Z, Zhang D. The effect of nicotine on RNA sequencing of mice spermatozoa. https://www.ncbi.nlm.nih.gov/geo/query/acc.cgi?acc=GSE155145. Accessed 6 May 2022.

58. Chen Y, Zheng Y, Gao Y, Lin Z, Yang S, Wang T, et al. Single-cell RNA-seq uncovers dynamic processes and critical regulators in mouse spermatogenesis. Cell Res. 2018;28:879–96.

59. Rinaldi VD, Donnard E, Gellatly K, Rasmussen M, Kucukural A, Yukselen O, et al. An atlas of cell types in the mouse epididymis and vas deferens. Elife. 2020;9:e55474.

60. Shi J, Fok KL, Dai P, Qiao F, Zhang M, Liu H, et al. Spatio-temporal landscape of mouse epididymal cells and specific mitochondria-rich segments defined by large-scale single-cell RNA-seq. Cell Discov. 2021;7:34.
